# Prediction of BMI at age 11 in a longitudinal sample of the Ulm Birth Cohort Study

**DOI:** 10.1371/journal.pone.0182338

**Published:** 2017-08-23

**Authors:** Hanna Christiansen, Stephanie Brandt, Viola Walter, Martin Wabitsch, Dietrich Rothenbacher, Hermann Brenner, Benno G. Schimmelmann, Oliver Hirsch

**Affiliations:** 1 Department of Clinical Child and Adolescent Psychology and Psychotherapy, Philipps University, Marburg, Germany; 2 University Medical Center Ulm, Department of Pediatrics and Adolescent Medicine, Division of Pediatric Endocrinology and Diabetes, Ulm, Germany; 3 German Cancer Research Center (DKFZ), Division of Clinical Epidemiology and Aging Research, Heidelberg, Germany; 4 Institute of Epidemiology and Medical Biometry, Ulm University, Ulm, Germany; 5 University Hospital of Child and Adolescent Psychiatry, University of Bern, Bern, Switzerland; 6 Department of Child and Adolescent Psychiatry, University Hospital Hamburg-Eppendorf, Hamburg, Germany; 7 FOM University of Applied Sciences, Siegen, Germany; Yale School of Public Health, UNITED STATES

## Abstract

Obesity is one of the greatest public health challenges in the world with childhood prevalence rates between 20–26% and numerous associated health risks. The aim of the current study was to analyze the 11-year follow-up data of the Ulm Birth Cohort Study (UBCS), to identify whether abnormal eating behavior patterns, especially restrained eating, predict body mass index (BMI) at 11 years of age and to explore other factors known to be longitudinally associated with it. Of the original UBCS, n = 422 children (~ 40% of the original sample) and their parents participated in the 11-year follow-up. BMI at age 8 and 11 as well as information on restrained eating, psychological problems, depressive symptoms, lifestyle, and IQ at age 8 were assessed. Partial Least Squares Structural Equation Modeling (PLS-SEM) was used to predict children’s BMI scores at age 11. PLS-SEM explained 68% of the variance of BMI at age 11, with BMI at age 8 being the most important predictor. Restrained eating, via BMI at age 8 as well as parental BMI, had further weak associations with BMI at age 11; no other predictor was statistically significant. Since established overweight at age 8 already predicts BMI scores at age 11 longitudinally, obesity interventions should be implemented in early childhood.

## Introduction

Childhood obesity prevalence rates range worldwide between 6–27% for males and 5–17% for females [1, 2]. Obesity is one of the greatest public health challenges in the world [1] with numerous associated physical health risks (among others e.g. cardiovascular and endocrinological diseases, type-2 diabetes, pubertas praecox in females and tarda in males, sleep disorders, and orthopedic problems; for a review see [1]). An association between obesity and psychological impairments, such as increased general psychopathology, and specifically depression, anxiety, increased stress, and overall reduced quality of life, has also been reported in different studies [3, 4].

With respect to risk factors for obesity, the following have been identified in several reviews and longitudinal studies: crossing the 85th body mass index (BMI) percentile in the first two years of life [5–7], increase in BMI at ages 7–9 [8, 9], low parental educational attainment and low socio-economic status (SES) [5, 10, 11], parental obesity [5, 10, 12–14], ethnic background [5, 10, 15], sugary beverage consumption [16], and a sedentary lifestyle [10]. Psychological risk factors have also been established such as depressive symptoms and other negative emotional states, low self-esteem, physical appearance [17], as well as abnormal eating patterns (i. e. restrained and emotional eating (overviews in: [18–21]).

The Ulm Birth Cohort Study (UCBS) is a population-based prospective study that has, beside other aims [22], assessed the development of BMI as well as different associated risk factors for over eleven years (2000 to 2011; for details see [23]). In a prior study, based on UBCS data, developmental pathways of obesity were established with a significant link between restraint eating and BMI in 8 year old children. Children restraining their food intake were more overweight than children who did not [24] which is in line with other studies [25–28]. The question of the current study is whether this established link at age eight also has associations with BMI development longitudinally. The aim of the current study is thus to analyze the 11-year follow-up data and (a) to identify whether abnormal eating behavior patterns, especially restrained eating, assessed at the age of 8 predict BMI at 11 years of age and (b) to explore whether other factors that have been identified as significant predictors in the literature (i. e. parental BMI, socio-economic factors, physical activity, general child psychopathology and specifically depressive symptoms; see above) are longitudinally associated with BMI at age 11.

## Methods

### Subjects & procedure

This study is part of the 11-year follow-up of the UBCS. For this prospective cohort study all women admitted to the Department of Gynaecology and Obstetrics at the University of Ulm, Germany, between November 2000 and 2001 to give birth to a baby were recruited (n = 1593). Inclusion criteria: understanding of the German, Turkish or Russian language, pregnancy duration of at least 32 weeks, birth weight of the newborn > 2000g, no transfer of the newborn to the Department of Paediatrics immediately after delivery for medical reasons, and mother staying at the postnatal ward after delivery. A total of 1.045 mothers fulfilled the inclusion criteria and participated in the study. Regular follow-up investigations have been conducted until children’s age six. Further details of the study design have been described elsewhere [22, 23].

In addition, an 8-year follow-up of the UBCS was performed. All participants of the UBCS who participated at the 6-year follow-up (n = 629; 60% from the original cohort) received a parent questionnaire and were invited to an ambulant clinical examination at the outpatient clinic of the Division of Pediatric Endocrinology and Diabetes in the Department of Pediatrics and Adolescent Medicine at the University of Ulm. At the 8-year follow-up, the parent questionnaire response rate was slightly higher with 60.7% of the original cohort (n = 634) and the participation rate of physical and psychological examinations was 51.3% (n = 536).

**Fig 1:**
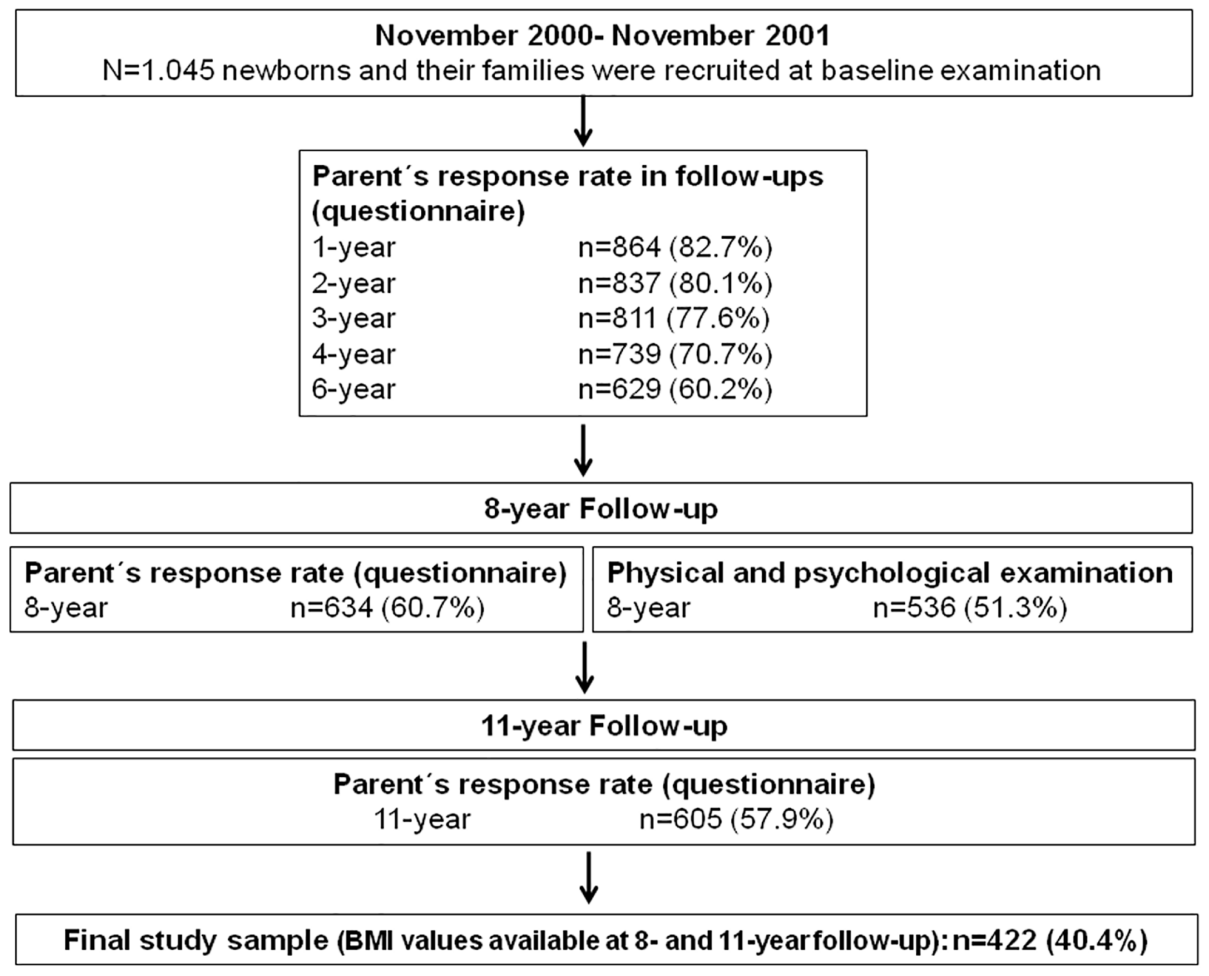
Study flow-chart.

At a child´s age of 11 years, an 11-year follow-up investigation was performed. All participants of the UBCS who participated at the 8-year follow-up received a parent questionnaire by mail; the response rate was 57.9% of the original cohort (n = 605). For the current study data from 422 children, of whom BMI data were available at ages 8 and 11, were analyzed (see Fig 1 for an overview of the whole study). Parents answered questions about current weight, the general health of their child and of family members, physical activity habits, dietary habits, media consumption, home situation and their child's wellbeing. Children completed a questionnaire including questions about physical activity habits, dietary habits, media consumption and child´s wellbeing. Participation was voluntary and written informed consent was obtained from all parents and oral assent from children. The study was approved by the Ethics Boards of the Universities Ulm and Heidelberg and by the Physicians’ Boards of the states Baden-Wuerttemberg and Bavaria, Germany.

### Measures

#### BMI at the 8- and 11-year follow-up of the UBCS

To establish under-/normal weight, overweight and obesity, the age standardized BMI was used [29]. At the 8-year follow-up, body height was measured three times, freestanding without shoes with a stadiometer fixed to the wall. Looking straight ahead, the Frankfurt line (virtual connection between top margin of the earhole exit to lower margin of the orbita) was kept horizontal during measurement. The mean of the three repeated measurements was calculated and taken as final body height. Body weight was assessed with a calibrated Seca-scale. Weight was measured when children were wearing their underwear only. BMI at age 8 was then calculated using the children’s weight (in kg) and height (in m) (BMI = kg/m^2^). At the 11-year follow-up current weight and height of the children were reported by the parents and used to calculate BMI values. It was demonstrated that parent-reported BMI and measured BMI lead to discrepant classifications in just 6% of children >10 years [30], and BMI self-report has also been successfully used in other studies as well [26].

As recommended by the World Health Organization (WHO), underweight, normal weight, and overweight were defined according to age specific BMI norms for boys and girls separately, with values lower than 1 standard deviation (SD) below the median defined as underweight and values higher than 1 SD above the median defined as overweight [31].

#### Eating Pattern Inventory for Children (EPI-C) at the 8- year follow-up of the UBCS

The EPI-C [32] investigates psychological dimensions of eating behavior as well as thoughts concerning weight in children aged ten years and older. The final version of this self-report questionnaire consists of 20 items that load on four dimensions (dietary restraint, external eating, parental pressure to eat, emotional eating), explaining a total of 62% of the variance. Factor 1 consists of eight items and explains 29.0% of total variance. It comprises affective, cognitive, and behavioral aspects of dietary restraint such as fear of becoming overweight, concerns about food and weight and actual restrictions in eating. These three aspects form one single inseparable factor. Factor 2 consists of five items related to external eating (eating in response to food-related stimuli regardless of internal states of hunger and satiety as well as constant feelings of hunger) and explains 17.9% of total variance. Factor 3 consists of three items expressing parental pressure to eat and explains 8.7% of total variance. Factor 4 consists of 4 items related to emotional eating, i.e., eating as a form of coping with emotional distress (explaining 6.4% of variance) [32]. Items are answered on a 4-point Likert scale: 1 = *not at all*, 2 = *little*, 3 = *mostly*, and 4 = *totally*. Internal consistency is satisfactory, with Cronbach’s α being .93 for dietary restraint, .80 for emotional eating, .74 for external eating, and .72 for parental pressure to eat [32]. In our own study, a confirmatory factor analysis in second-grade students (N = 521) supported the hypothesized structure of the EPI-C with the following Cronbach’s α coefficients: .87 for dietary restraint, .71 for external eating, .60 for parental pressure to eat, and .79 for emotional eating. Regarding validity, children with overweight reported significantly more dietary restraint than children with underweight or normal weight. Boys reported a significantly higher parental pressure to eat than girls. Children with restrained eating patterns had a significantly higher body weight than all other subgroups and children with emotional and external eating patterns had a higher body weight than children with external eating patterns [24]. When filling in the EPI-C the current sample was on average 8.25 years old (SD 0.17); the EPI-C was not filled out again at the 11 year follow-up, but data from the 8 year follow-up was used for this study.

#### Child Behavior Checklist (CBCL) at the 8- year follow-up of the UBCS

The CBCL 4–18 [33, 34] is a questionnaire to screen for emotional, behavioral, and social problems. It assesses psychological problems in children aged 4 to 18 years. In the current study, the parent version was used that is to be rated on a three point Likert scale (*0 = not at all; 1 = a little; 2 = a lot*). The eight included problem scales (anxiety/depression, withdrawal/depression, somatic complaints, social problems, cognitive-/sleep-/repetitive problems, attention problems, defiant and aggressive behavior) are summed up to three superior scales (total score, internalizing and externalizing problems). Internalizing behaviors are ones where children direct emotions and feelings inward. These are somatic complaints, anxious/depressed behavior, and withdrawn behavior. Somatic problems consist of feeling tired, aches, nausea, vomiting, headaches, dizziness and complaints about skin, stomach or eye problems. Withdrawn behaviors are measured by questions addressing social withdrawal, shyness, and sadness. Symptoms of anxiety/depression are addressed by questions regarding crying, fear, loneliness, nervousness, worthlessness, and worries. When emotional problems are directed outward, they are manifested as aggressive or delinquent behavior and are labeled externalizing behaviors. Delinquent externalizing behaviors assessed by the CBCL include for example cheating, lying, setting fires, stealing, and vandalism. Several types of aggressive behaviors are measured, like arguing, screaming, attention-seeking, teasing, being demanding, threatening behavior and displaying a temper.

All scales possess satisfying psychometric properties (α > .80), and norms adjusted for age and gender are provided.

#### The Depression Inventory for Children (DIKJ) at the 8- year follow-up of the UBCS

The DIKJ [35] is a self-rating questionnaire and assesses depressive symptoms of children aged 8 to 16 years based on the DSM-IV/5 [36]. It measures the degree of a depressive disorder in childhood and adolescence with 29 items covering areas like anhedonia, social withdrawal, and somatic symptoms, but also epiphenomena like difficulties at school on a three-point scale. The DIKJ can be used for therapy control as it is sensitive to change. Internal consistency is highly satisfactory with α .92 for a clinical and .87 for a representative sample. The total score was able to discriminate between children and adolescents with and without depression. Correlations with other instruments were in the expected directions with most validity coefficients around .30. Norms are provided adjusted for age and gender and are based on 3395 children and adolescents.

#### Assessment of intelligence quotient (IQ) at the 8-year follow-up of the UBCS

Raven´s Coloured Progressive Matrices (CPM) were used for the language free assessment of basic intelligence functions [37]. It can be applied in children from ages 3 years and 9 months to 11 years and 8 months and consists of three sets of 12 tasks each so that a maximum raw score of 36 is possible. Each task consists of colored incomplete geometrical shapes or figures to be correctly completed out of a set of 6 alternatives. The three sets offer the participants three different possibilities to develop consistent methods of analytical thinking. The CPM were developed to assess intellectual abilities in subjects who are not able to speak and understand German, who have physical disabilities, aphasia, cerebral paresis, who are deaf or who are mentally handicapped. Age norms are available in half year distances. Split-half reliability lies between r = .85 and .90, retest reliability after two weeks was between r = .86 and .90. It was shown that the items of the CPM load on a factor “simultaneous processing” with loadings ranging from .75 to .85.

### Statistical analysis

We performed Partial Least Squares Structural Equation Modeling (PLS-SEM) predicting children´s BMI at 11 years. PLS-SEM is a variant of structural equation modeling which uses an ordinary least squares regression-based method (OLS) in contrast to the maximum likelihood estimation procedure in covariance-based structural equation modeling. PLS-SEM is a variance-based approach. PLS-SEM should be used in situations where theory is less developed and the aim is to predict and explain target constructs. It works with small sample sizes, complex models and makes almost no assumptions about the level of measurement of the data [38]. For model estimation we used the path weighting scheme which standardizes the included variables. To obtain tests of significance for path coefficients and outer loadings of variables forming latent constructs we performed bootstrapping with 5000 samples of n = 422 each. The resulting t-values were then tested for significance. We considered a p-value of ≤.05 to be significant. To evaluate the structural model, the coefficient of determination R^2^ can be used. Values of at least .75, .50, and .25 for endogenous latent variables can be considered substantial, moderate, or weak, respectively [39]. The effect size f^2^ demonstrates whether an exogenous construct has a substantive impact on an endogenous construct. Values of at least .02, .15, and .35 represent small, medium, and large effects, respectively [40]. To evaluate the measurement model, the outer (factor) loadings of variables on their respective latent constructs should be at least .70. Factor loadings can only be calculated if more than one manifest variable represents a latent construct. The internal consistency of latent constructs with more than one manifest variable is measured by the composite reliability. Values of .60 to .70 are acceptable in exploratory research [41]. The average variance extracted (AVE) shows the proportion of variance the constructs explain in its indicators. It is equivalent to the communality in factor analysis and can be regarded as a measure of convergent validity. Heterogeneity was examined with the FIMIX-PLS algorithm [42], multicollinearity was present if indicators had a variance inflation factor (VIF) > 5 [38]. A limitation of this method is that there is no global goodness-of-fit criterion.

**Fig 2:**
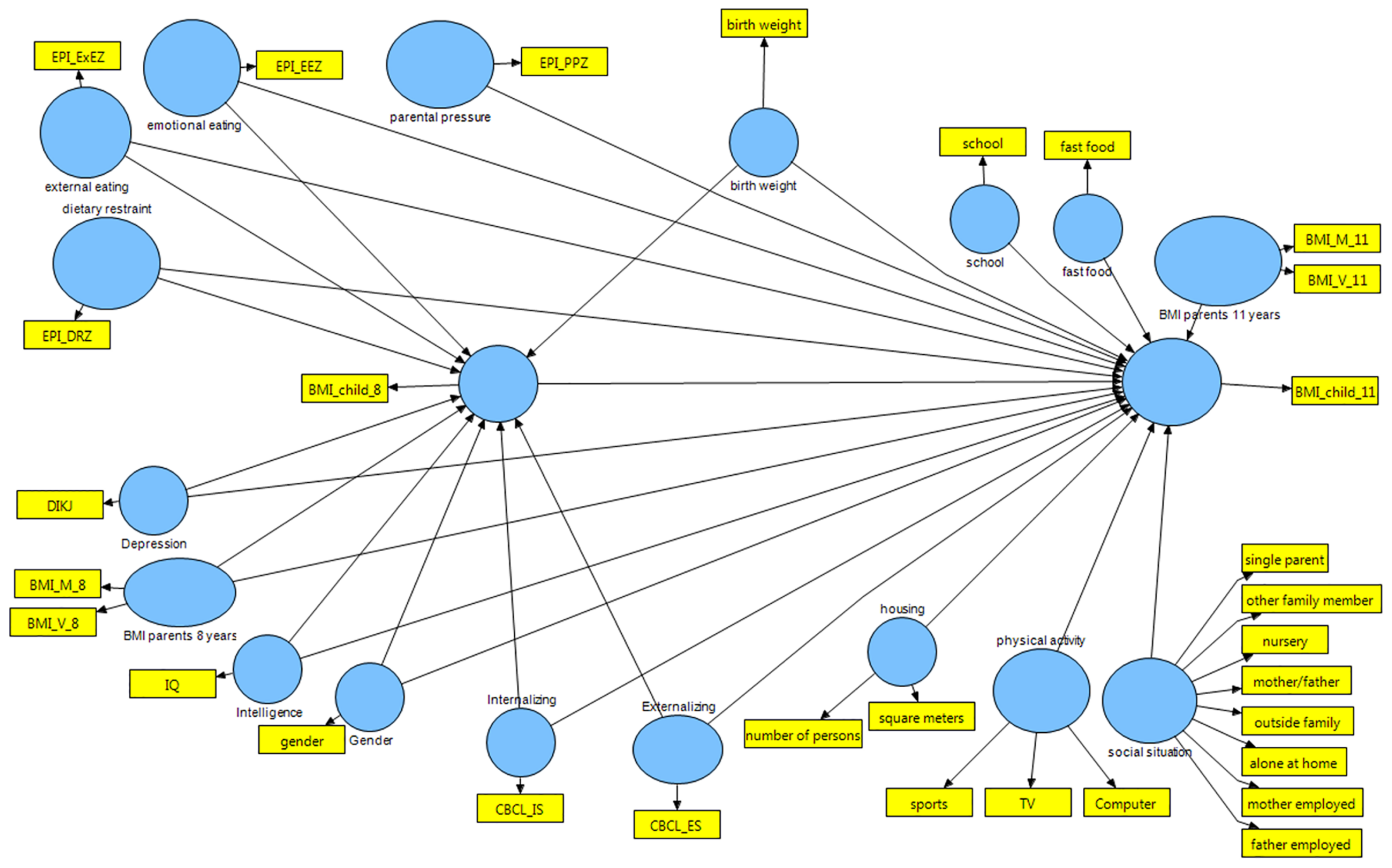
Partial Least Squares Structural Equation Model (PLS-SEM) predicting children´s BMI at 11 years.

As predictors we used the EPI-C scales external eating, emotional eating, dietary restraint, and parental pressure to eat of which the first three are known to have an association with children´s BMI at age 8 [24]. We further utilized depressive symptoms, birth weight, parental BMI at children´s age 8 and 11, IQ, gender, CBCL internalizing and externalizing disorders, housing situation (composed of number of persons living together, square meters of apartment/house, smoking in apartment/house yes/no), physical activity (composed of five-point ratings regarding sports, TV consumption, and playing video game consoles), social situation (composed of dichotomous variables single parent, being cared for by another family member, attending a nursery, being cared for by mother or father, being cared for by people outside the family, being alone at home, mother employed, father employed), a four-point rating regarding fast food consumption, and school attendance as predictors of children´s BMI at age 11. The complete model is depicted in Fig 2. All calculations were done with the program SmartPLS 2.0.M3 (http://www.smartpls.de/).

## Results

Table 1 displays the sample characteristics of participating children in the total sample stratified according to gender.

**Table 1:** Sample characteristics of participating children in the total sample and stratified according to gender with means and standard deviations (SD) of metric variables and absolute numbers and percent of categorical variables in parentheses.

	Total sample	Boys	Girls
	N = 422	n = 208 (49.3%)	n = 214 (50.7%)
Age (years), mean (SD)	11.03 (0.07)	11.04 (0.07)	11.02 (0.06)
Birth weight (g), mean (SD)	3405.6 (494.7)	3507.6 (493.7)	3306.4 (476.3)
BMI 11 years (kg/m^2^)	17.44 (2.56)	17.63 (2.68)	17.26 (2.43)
underweight n (%)	80 (19.0%)	34 (16.3%)	46 (21.5%)
normal weight	291 (69.0%)	143 (68.8%)	148 (69.2%)
overweight	51 (12.0%)	31 (14.9%)	20 (9.3%)
BMI 8 years (kg/m^2^)	16.13 (2.05)	16.24 (2.13)	16.02 (1.97)
underweight n (%)	77 (18.2%)	36 (17.3%)	41 (19.2%)
normal weight	284 (67.3%)	139 (66.8%)	145 (67.8%)
overweight	61 (14.5%)	33 (15.9%)	28 (13.0%)
Mother´s BMI at child´s age 8 (kg/m^2^)	24.50 (4.56)	24.43 (4.71)	24.56 (4.43)
Father´s BMI at child´s age 8 (kg/m^2^)	26.24 (3.33)	26.24 (3.45)	26.24 (3.21)
Mother´s BMI at child´s age 11 (kg/m^2^)	23.84 (4.03)	23.84 (4.28)	23.84 (3.79)
Father´s BMI at child´s age 11 (kg/m^2^)	25.92 (3.21)	25.95 (3.26)	25.89 (3.17)
IQ at age 8 (score)	114.03 (10.58)	114.04 (10.62)	114.01 (10.57)
Depressive symptoms (DIKJ) (raw score)	8.09 (4.63)	8.34 (4.54)	7.84 (4.72)
CBCL Internalizing (T score)	55.27 (4.88)	55.11 (4.77)	55.43 (4.99)
CBCL Externalizing (T score)	53.57 (4.28)	53.55 (4.57)	53.60 (3.98)
EPI-C dietary restraint (z score)	0.00 (1.00)	-0.04 (0.96)	0.04 (1.01)
EPI-C external eating (z score)	0.00 (1.00)	-0.09 (0.95)	0.04 (1.05)
EPI-C parental pressure (z score)	0.00 (1.00)	0.09 (1.07)	-0.12 (0.96)
EPI-C emotional eating (z score)	0.00 (1.00)	-0.06 (1.01)	0.04 (1.02)
Number of persons in apartment/house	4.11 (0.87)	4.13 (0.93)	4.08 (0.80)
Square meters of apartment/house	139.77 (41.03)	136.48 (38.22)	142.96 (43.43)

Table 2 shows sample characteristics for socio-economic and questionnaire variables, not stratified according to gender.

**Table 2:** Sample characteristics of participating children in the total sample for socio-economic and questionnaire variables targeting physical activity, food intake, TV and computer consumption at age 11, not stratified according to gender; missings per row were very low (0.2–1.9%) and not reported in detail.

Latent construct	Variables	Categories	n (%)
**School situation**	Attendance of school	Primary school	15 (3.6%)
		Secondary modern school	21 (5.0%)
		Junior high school	121 (28.7%)
		Grammar school	254 (60.2%)
		Comprehensive school	4 (0.9%)
**Leisure time activities**	Children’s self-reported fast food consumption	Once a week	49 (11.6%)
		2–3 times per month	7 (1.7%)
		Once a month or less	362 (85.8%)
	Physical activity	Every day	119 (28.2%)
		3–5 times per week	202 (47.9%)
		1–2 times per week	91 (21.6%)
		1–2 times per month	4 (0.9%)
		Never	2 (0.5%)
	TV consumption	Never	75 (17.8%)
		30 Min. per day	176 (41.7%)
		1–2 h per day	154 (36.5%)
		3–4 h per day	8 (1.9%)
		>4 h per day	1 (0.2%)
	Computer consumption	Never	167 (39.6%)
		30 Min. per day	208 (49.3%)
		1–2 h per day	38 (9.0%)
		3–4 h per day	1 (0.2%)
		>4 h per day	1 (0.2%)
**Social situation**	Single parent	Yes	34 (8.1%)
		No	387 (91.7%)
	Cared for by other family member	Yes	59 (14.0%)
		No	361 (85.5%)
	Attends nursery	Yes	27 (6.4%)
		No	393 (93.1%)
	Cared for by Mother/father	Yes	395 (93.6%)
		No	25 (5.9%)
	Cared for outside family	Yes	11 (2.6%)
		No	409 (96.9%)
	Alone at home	Yes	5 (1.2%)
		No	415 (98.3%)
	Mother employed	Yes	334 (79.1%)
		No	84 (19.9%)
	Father employed	Yes	407 (96.4%)
		No	8 (1.9%)

The FIMIX-PLS algorithm indicated that there is no unobserved heterogeneity in our data. First, the quality of the structural model was examined. Table 3 depicts the path coefficients of the original sample, the means and standard errors (SE) of the bootstrap samples, the t-values and the resulting p-values.

**Table 3:** Path coefficients of latent constructs predicting either children´s BMI at age 8 or children´s BMI at age 11. Significant t values after bootstrapping are printed in bold.

	Original Sample (O)	Sample Mean (M)	SE	t value	p
**Predictors of BMI at 11 years**					
BMI 8 years	0.80	0.80	0.03	**26.07**	**<.001**
BMI parents 11 years	0.07	0.07	0.03	**2.37**	**.02**
Birth weight	-0.013	-0.014	0.028	0.45	.65
EPI-C dietary restraint	-0.063	-0.066	0.035	1.81	.07
EPI-C external eating	-0.058	-0.056	0.03	1.80	.07
EPI-C parental pressure	-0.04	-0.036	0.03	1.33	.18
IQ	-0.037	-0.038	0.029	1.27	.21
Physical activity	0.042	0.043	0.033	1.25	.21
Social situation	0.049	0.065	0.04	1.23	.22
BMI parents 8 years	-0.03	-0.03	0.03	1.00	.32
Fast food consumption	-0.032	-0.031	0.032	0.99	.32
Depressive symptoms	0.03	0.03	0032	0.97	.33
CBCL Internalizing Disorders	-0.027	-0.027	0.035	0.76	.45
Gender	-0.018	-0.019	0.028	0.66	.51
Housing	-0.02	0.00	0.05	0.43	.67
EPI-C emotional eating	0.011	0.011	0.031	0.35	.73
CBCL Externalizing Disorders	0.003	0.002	0.029	0.09	.93
School	0.003	0.002	0.034	0.08	.94
**Predictors of BMI at 8 years**					
EPI-C dietary restraint	0.42	0.42	0.05	**8.47**	**<.001**
Birth weight	0.19	0.19	0.05	**4.08**	**<.001**
BMI parents 8 years	0.13	0.14	0.05	**3.00**	**.002**
EPI-C emotional eating	-0.08	-0.08	0.05	1.55	.12
Gender	-0.033	-0.033	0.044	0.76	.45
IQ	-0.095	-0.094	0.05	1.91	.06
CBCL Internalizing Disorders	0.073	0.071	0.053	1.37	.17
Depression	-0.043	-0.044	0.042	1.03	.30
EPI-C external eating	-0.04	-0.04	0.05	0.80	.42
CBCL Externalizing Disorders	-0.022	-0.020	0.05	0.44	.66

The R^2^ for children´s BMI at age 11 was .684 while the R^2^ for children´s BMI at age 8 was .256.

As seen in Table 3, the largest predictor for children´s BMI at age 11 was children´s BMI at age 8. The value of the effect size f^2^ amounts to 1.45 and signals a very large association. A small association exists between parental and children´s BMI at children’s age 11 (f^2^ = 0.013). The EPI-C subscale restraint eating had a substantial total association with children´s BMI at age 11. With reference to Fig 2 and Table 2, the total effect of .27 is calculated as follows: .42 (path coefficient dietary restraint on BMI 8 years) * .80 (path coefficient BMI 8 years on BMI 11 years) + (-.063; path coefficient dietary restraint on BMI 11 years). All other predictors for children´s BMI at age 11 were not significant.

Significant predictors for children´s BMI at age 8 were dietary restraint in EPI-C (f^2^ = 0.22, medium effect), parent´s BMI at children´s age 8 (f^2^ = 0.02, small effect) and birth weight (f^2^ = 0.02, small effect). All other predictors for children´s BMI at age 8 were not significant.

In the next step, the measurement model (outer model) was examined. None of the indicators had a VIF > 5, therefore no multicollinearity was found. Table 4 displays the factor loadings of the variables loading on their latent constructs in the original sample, the means and SE of the bootstrap samples, the t-values and the resulting p-values.

**Table 4:** Outer (factor) loadings of variables on their latent constructs. Significant t values after bootstrapping are printed in bold.

	Original Sample (O)	Sample Mean (M)	SE	t value	p
**Social situation**					
Single parent	-0.057	-0.038	0.25	0.23	.82
Other family members	-0.14	-0.07	0.29	0.49	.62
Nursery	-0.29	-0.15	0.31	0.93	.35
Mother/father	0.51	0.32	0.36	1.45	.15
Outside family	0.30	0.21	0.22	1.36	.18
Alone at home	0.19	0.14	0.27	0.69	.49
Mother employed	**0.62**	0.42	0.27	**2.32**	**.02**
Father employed	0.12	0.07	0.34	0.36	.72
**Parent´s BMI at children´s age 11**					
BMI father	**0.80**	0.80	0.07	**11.97**	**<.001**
BMI mother	**0.76**	0.76	0.07	**10.23**	**<.001**
**Parent´s BMI at children´s age 8**					
BMI mother	0.36	0.34	0.29	1.26	.21
BMI father	**0.93**	0.88	0.16	**5.87**	**<.001**
**Housing**					
Number of persons living together	0.21	0.21	0.38	0.55	.58
Square meters	**0.75**	0.63	0.32	**2.35**	**.02**
**Physical activity**					
Sports	0.01	0.03	0.32	0.04	.97
TV consumption	**0.96**	0.84	0.28	**3.49**	**.001**
Computer consumption	0.49	0.42	0.25	1.95	.052

Table 3 demonstrates that of the latent construct “social situation” the variable “mother employed” has a significant loading of .62. The composite reliability of this construct is .18 and the AVE is .11. This is due to the skewed distribution of the majority of the variables involved. Both parents’ BMI variables at children´s age 11 load high on their latent construct which has a satisfactory composite reliability of .76 and an AVE of .61. On the latent construct “parental BMI at children´s age 8” father´s BMI has a significant loading of .93. The construct has a composite reliability of .62 and an AVE of .50. The manifest variable “square meters of apartment/house” has a significant loading on the latent construct “housing” while the loading of “smoking in apartment/house” misses significance. The composite reliability of this construct is .57 and therefore still acceptable, the AVE is .35. A significant positive loading on the construct “physical activity” is reached by the variable “TV consumption”. The loading of “computer consumption” misses statistical significance. The composite reliability of this construct is .54 and slightly under the recommended cut-off of .60, the AVE is .39.

## Discussion

In this cohort study involving a large number of children we were able to demonstrate significant longitudinal associations of different covariates with children’s BMI at age eleven with 68% variance explained. The most important predictor for children´s BMI at age 11 was children´s BMI at age 8. The value of the effect size signaled a very large association. The usefulness of including the BMI at age 8 as a predictor for BMI at age 11 is confirmed by two large German studies with about 3500 and more than 17500 partcipants. It was demonstrated that the largest increases in BMI occurred at ages 7 to 9. Consequently, BMI at this age is an important predictor for BMI at later ages [8, 9]. This confirms prior studies that identified early overweight as a relevant risk factor for obesity in later childhood [4, 6, 7, 10, 43]. A small association existed between parental BMI and children´s BMI at age 11, an observation also in line with other reported research findings in the field [4, 11–15]. However, the EPI-C subscale restraint eating had a substantial total effect via children´s BMI at age 8 on children´s BMI at age 11. Thus, abnormal eating behaviors that are associated with an increase of BMI at the age of 8 show longitudinal associations, though not directly but mediated through prior BMI. The likely explanation is that children who are overweight restrain their food intake, most likely influenced by parents. Similar effects have also been demonstrated in three other longitudinal studies. In the study by Forrester-Knauss et al. [26], with N = 428 children assessed as pre-schoolers and pre-adolescents, restrained eating at the age of 12 years was significantly predicted by BMI at the age of 5 years as well as by BMI change over time. Munkholm and colleagues [27, 28] analyzed data of the Copenhagen Child Cohort (CCC2000) that assessed restrained, emotional and external eating with the EPI-C in a general population sample of 11–12 year olds. Restrained eating was significantly associated with overweight, body dissatisfaction and emotional disorders in both genders. Further, overeating patterns at the age of 5–7 years prospectively predicted restrained eating in preadolescence (OR 2.77) and overweight at 11–12 years (OR 4.79). Those longitudinal studies confirm others that demonstrated an association of restrained food intake with higher BMI scores [18–21], and support thus the assumption that higher weight and restrained eating lead to a vicious circle that continues to increase weight. These findings are in line with studies demonstrating that highly restrained eaters have a higher chance of being in higher BMI trajectories, whereas other factors such as emotional and external eating were unrelated to BMI [24]. No other of the investigated predictors was statistically significantly associated with children´s BMI at age 11, replicating the findings of the other three longitudinal studies on restrained eating and BMI over time [26–28], though Munkholm et al. [27] also identified a low annual household income as a further strong explanatory factor of BMI increase (OR 2.06), and Forrester-Knauss et al. [26] identified an association of body self-esteem and longitudinal BMI development.

Prediction of BMI at age 8 was weak with substantially less variance explained (25.6%). BMI at age 8 was predicted by a higher dietary restraint assessed with the EPI-C, replicating results of other studies in the field [26–28], by a higher parental BMI at child’s age 8, and by a higher birth weight. Regarding the measurement model we conclude that the latent construct “social situation” did not satisfy statistical quality criteria while quality criteria of the other latent constructs, such as “parental BMI at age 8”, “housing”, and “physical activity”, were fulfilled. PLS-SEM proved to be a valuable data analysis strategy for our research question with prior BMI being the most significant predictor of later BMI, even though the prediction of BMI at an earlier age seems to be less reliable with respect to the amount of variance explained. Overall, the main risk factors identified at age 8 concur with the ones of prior research in the field [4, 6, 15, 19, 21, 24, 43].

There are some limitations to be considered. The participation rate in our follow-up sample was significantly lower than in the initial sample, although response rates such as ours are not unusual in birth cohort studies with long-term follow-ups [44]. Bearing this limitation in mind, this is still a large sample for a longitudinal study over 11 years. Also, our study was conducted in a single urban area and is not representative of the German population. When we compare the follow-up sample included in this study with the complete sample at baseline it is evident that families with higher duration of school education were more likely to participate. These limitations restrain the generalizability of our results. In addition, abnormal eating behaviors and BMI were both assessed at age 8, therefore reverse causation regarding BMI and dietary restraint cannot be excluded. Children´s BMI at age 11 was reported by their parents and was not directly measured, though other studies have also successfully used self-reported BMI which proved to be fairly reliable [26]. To establish possible cause and effect paths, longer longitudinal studies are required. Nevertheless, since other studies in the field point to the relevance of abnormal eating patterns, especially restrained eating for increased BMI [18–21, 45, 46], these patterns should be a target for intervention programs.

With respect to the relevance of the results obtained for the broader field of obesity prevention and intervention strategies one could argue that an implementation at a very early age should be recommended [26]. There is a significant association of higher birth weight and BMI at age 8 in our study and the children at age 8 in our study already showed abnormal eating patterns, especially restrained eating and this is paradoxically associated with the failure to control eating [45, 47] and associated with higher BMI at age 8 which in turn predicted BMI at age 11. A current study analyzed associations of personality factors and BMI and demonstrated that high extraversion scores at age 5 years significantly predicted higher BMI and restrained eating at age 12 [48]. An experimental study on restrained eating and food intake revealed that restrained eaters successfully restrain their food intake when this is task relevant, but show a strong food approach when they were in a good mood and food was task irrelevant [49]. Thus, effective interventions need to take effect before children develop overweight or obesity, and abnormal eating patterns, and should consider other psychological factors such as personality and task relevance. Other research on early intervention has demonstrated that the vast majority of interventions for children show the largest benefit in the first three years of life [46]. Further, early care and education (ECE) has been identified as a priority setting for obesity prevention (http://www.cdc.gov/obesity/strategies/childcareece.html). According to these best practice recommendations, standards for obesity prevention have been established, addressing nutrition, infant feeding, physical activity and screen time, that involve parents and care takers. This is especially important, since parental BMI is a significant predictor for children’s BMI [10, 14, 16–18], as shown in our study as well. Ideally, midwifes and day cares/play schools should be involved in such interventions, since this would allow implementation on a universal level with a low-threshold that could reach the majority of families without stigmatization. Such strategies are also recommended by the Centers for Disease Control and Prevention (http://www.cdc.gov/obesity/strategies/index.html) and are a crucial task for the 21^st^ century, since curative interventions only reach a minority of affected people and can be considered a drop in the ocean with prevalence rates of obesity of up to 26% [1, 2].

## Conclusion

Obesity is one of the world’s greatest public health challenges. Higher birth weight and restrained eating is associated with overweight at age 8 and continues to exert its influence via BMI at age 11. Interventions for obesity should be implemented at a very early age [26], since established overweight longitudinally predicts BMI scores. It is much harder to reduce overweight than to prevent its onset in the first place.

## References

[1] Centers for Disease Control and Prevention. School health guidelines to promote healthy eating and physical activity. MMWR Recomm Rep. 2011;60(RR-5):1–76. Epub 2011/09/16. . 21918496

[2] Wijnhoven TM, van Raaij JM, Spinelli A, Rito AI, Hovengen R, Kunesova M, et al WHO European Childhood Obesity Surveillance Initiative 2008: weight, height and body mass index in 6-9-year-old children. Pediatr Obes. 2013;8(2):79–97. Epub 2012/09/25. doi: 10.1111/j.2047-6310.2012.00090.x . 23001989 10.1111/j.2047-6310.2012.00090.x

[3] Doyle AC, le Grange D, Goldschmidt A, Wilfley DE. Psychosocial and physical impairment in overweight adolescents at high risk for eating disorders. Obesity (Silver Spring). 2007;15(1):145–54. doi: 10.1038/oby.2007.515 . 17228042 10.1038/oby.2007.515

[4] Luppino FS, de Wit LM, Bouvy PF, Stijnen T, Cuijpers P, Penninx BW, et al Overweight, obesity, and depression: a systematic review and meta-analysis of longitudinal studies. Arch Gen Psychiatry. 2010;67(3):220–9. doi: 10.1001/archgenpsychiatry.2010.2 . 20194822 10.1001/archgenpsychiatry.2010.2

[5] Flores G, Lin H. Factors predicting severe childhood obesity in kindergarteners. Int J Obes (Lond). 2013;37(1):31–9. Epub 2012/11/14. doi: 10.1038/ijo.2012.168 . 23147114 10.1038/ijo.2012.168

[6] Rifas-Shiman SL, Gillman MW, Oken E, Kleinman K, Taveras EM. Similarity of the CDC and WHO weight-for-length growth charts in predicting risk of obesity at age 5 years. Obesity (Silver Spring). 2012;20(6):1261–5. Epub 2011/12/14. doi: 10.1038/oby.2011.350 . 22158005 10.1038/oby.2011.350

[7] Dubois L, Girard M. Early determinants of overweight at 4.5 years in a population-based longitudinal study. Int J Obes (Lond). 2006;30(4):610–7. doi: 10.1038/sj.ijo.0803141 . 16570091 10.1038/sj.ijo.0803141

[8] Hoffmann SW, Ulrich R, Simon P. Refined analysis of the critical age ranges of childhood overweight: implications for primary prevention. Obesity (Silver Spring). 2012;20(10):2151–4. doi: 10.1038/oby.2012.172 . 22714087 10.1038/oby.2012.172

[9] Funk MB, Bausback-Schomakers S, Hanschmann K.M., Gerhards B, Kuhn K, Krackhardt B. Gewichtsentwicklung im frühen Grundschulalter. Prävalenz, Inzidenz und Risikofaktoren für Übergewicht und Adipositas. [Development of body weight in elementary school. Prevalence, incidence, and risk factors for overweight and adipositas.]. Bundesgesundheitsblatt—Gesundheitsforschung—Gesundheitsschutz. 2015;58(10):1110–7. 26285649 10.1007/s00103-015-2220-8

[10] Brophy S, Cooksey R, Gravenor MB, Mistry R, Thomas N, Lyons RA, et al Risk factors for childhood obesity at age 5: analysis of the millennium cohort study. BMC Public Health. 2009;9:467 Epub 2009/12/18. doi: 10.1186/1471-2458-9-467 ; 20015353 10.1186/1471-2458-9-467 PMC2803190

[11] Power C, Parsons T. Nutritional and other influences in childhood as predictors of adult obesity. Proc Nutr Soc. 2000;59(2):267–72. . 10946795 10.1017/s002966510000029x

[12] Keane E, Layte R, Harrington J, Kearney PM, Perry IJ. Measured parental weight status and familial socio-economic status correlates with childhood overweight and obesity at age 9. PLoS One. 2012;7(8):e43503 Epub 2012/08/23. doi: 10.1371/journal.pone.0043503 ; 22912886 10.1371/journal.pone.0043503 PMC3422292

[13] Olstad DL, McCargar L. Prevention of overweight and obesity in children under the age of 6 years. Appl Physiol Nutr Metab. 2009;34(4):551–70. Epub 2009/09/22. doi: 10.1139/H09-016 . 19767789 10.1139/H09-016

[14] Rooney BL, Mathiason MA, Schauberger CW. Predictors of obesity in childhood, adolescence, and adulthood in a birth cohort. Matern Child Health J. 2011;15(8):1166–75. Epub 2010/10/12. doi: 10.1007/s10995-010-0689-1 . 20927643 10.1007/s10995-010-0689-1

[15] Long JM, Mareno N, Shabo R, Wilson AH. Overweight and obesity among White, Black, and Mexican American children: implications for when to intervene. J Spec Pediatr Nurs. 2012;17(1):41–50. Epub 2011/12/23. doi: 10.1111/j.1744-6155.2011.00309.x . 22188271 10.1111/j.1744-6155.2011.00309.x

[16] Vartanian LR, Schwartz MB, Brownell KD. Effects of soft drink consumption on nutrition and health: a systematic review and meta-analysis. Am J Public Health. 2007;97(4):667–75. Epub 2007/03/03. doi: 10.2105/AJPH.2005.083782 ; 17329656 10.2105/AJPH.2005.083782 PMC1829363

[17] Incledon E, Wake M, Hay M. Psychological predictors of adiposity: systematic review of longitudinal studies. Int J Pediatr Obes. 2011;6(2–2):e1–11. doi: 10.3109/17477166.2010.549491 . 21247271 10.3109/17477166.2010.549491

[18] Braet C, Van Strien T. Assessment of emotional, externally induced and restrained eating behaviour in nine to twelve-year-old obese and non-obese children. Behav Res Ther. 1997;35(9):863–73. Epub 1997/09/23. . 9299807 10.1016/s0005-7967(97)00045-4

[19] Braet C, Claus L, Goossens L, Moens E, Van Vlierberghe L, Soetens B. Differences in eating style between overweight and normal-weight youngsters. J Health Psychol. 2008;13(6):733–43. Epub 2008/08/14. doi: 10.1177/1359105308093850 . 18697886 10.1177/1359105308093850

[20] Carnell S, Benson L, Pryor K, Driggin E. Appetitive traits from infancy to adolescence: Using behavioral and neural measures to investigate obesity risk. Physiol Behav. 2013 Epub 2013/03/06. doi: 10.1016/j.physbeh.2013.02.015 ; 23458627 10.1016/j.physbeh.2013.02.015 PMC3725261

[21] Snoek HM, Engels RC, van Strien T, Otten R. Emotional, external and restrained eating behaviour and BMI trajectories in adolescence. Appetite. 2013;67:81–7. Epub 2013/04/11. doi: 10.1016/j.appet.2013.03.014 . 23571047 10.1016/j.appet.2013.03.014

[22] Weyermann M, Rothenbacher D, Brenner H. Acquisition of Helicobacter pylori infection in early childhood: independent contributions of infected mothers, fathers, and siblings. Am J Gastroenterol. 2009;104(1):182–9. doi: 10.1038/ajg.2008.61 . 19098867 10.1038/ajg.2008.61

[23] Brandt S, Moss A, Koenig W, Rothenbacher D, Brenner H, Wabitsch M. Intrafamilial associations of cardiometabolic risk factors—results of the Ulm Birth Cohort Study. Atherosclerosis. 2015;240(1):174–83. doi: 10.1016/j.atherosclerosis.2015.02.045 . 25796035 10.1016/j.atherosclerosis.2015.02.045

[24] Hirsch O, Kluckner VJ, Brandt S, Moss A, Weck M, Florath I, et al Restrained and external-emotional eating patterns in young overweight children-results of the Ulm Birth Cohort Study. PLoS One. 2014;9(8):e105303 doi: 10.1371/journal.pone.0105303 ; 25141134 10.1371/journal.pone.0105303 PMC4139345

[25] Snoek HM, van Strien T, Janssens JM, Engels RC. Emotional, external, restrained eating and overweight in Dutch adolescents. Scand J Psychol. 2007;48(1):23–32. doi: 10.1111/j.1467-9450.2006.00568.x . 17257366 10.1111/j.1467-9450.2006.00568.x

[26] Forrester-Knauss C, Perren S, Alsaker FD. Does body mass index in childhood predict restraint eating in early adolescence? Appetite. 2012;59(3):921–6. doi: 10.1016/j.appet.2012.08.026 . 22983367 10.1016/j.appet.2012.08.026

[27] Munkholm A, Olsen EM, Rask CU, Clemmensen L, Rimvall MK, Jeppesen P, et al Early Predictors of Eating Problems in Preadolescence-A Prospective Birth Cohort Study. J Adolesc Health. 2016;58(5):533–42. doi: 10.1016/j.jadohealth.2016.01.006 . 27107908 10.1016/j.jadohealth.2016.01.006

[28] Munkholm A, Olsen EM, Rask CU, Clemmensen L, Rimvall MK, Jeppesen P, et al Eating behaviours in preadolescence are associated with body dissatisfaction and mental disorders—Results of the CCC2000 study. Appetite. 2016;101:46–54. doi: 10.1016/j.appet.2016.02.020 . 26896837 10.1016/j.appet.2016.02.020

[29] Knußmann R. Vergleichende Biologie des Menschen: Lehrbuch der Anthropologie und Humangenetik [Comparative Human Biology: Textbook of Anthropology and Human Genetics]. Stuttgart: Fischer; 1996.

[30] Skinner AC, Miles D, Perrin EM, Coyne-Beasley T, Ford C. Source of parental reports of child height and weight during phone interviews and influence on obesity prevalence estimates among children aged 3–17 years. Public Health Rep. 2013;128(1):46–53. doi: 10.1177/003335491312800107 ; 23277659 10.1177/003335491312800107 PMC3514720

[31] Kromeyer-Hauschild K, Wabitsch M, Kunze D, Geller F, Geiss HC, Hesse V. Perzentile für den Bodymass-Index für das Kindes- und Jungendalter unter Heranziehung verschiedener deutscher Stichproben. [Percentile scores for the body mass index in children and adolescents based on different German samples.]. Monatsschrift für Kinderheilkunde. 2001;149:807–18.

[32] Schacht M, Richter-Appelt H, Schulte-Markwort M, Hebebrand J, Schimmelmann BG. Eating Pattern Inventory for Children: a new self-rating questionnaire for preadolescents. J Clin Psychol. 2006;62(10):1259–73. Epub 2006/08/10. doi: 10.1002/jclp.20300 . 16897691 10.1002/jclp.20300

[33] Achenbach TM. Integrative guide for the 1991 CBCL/4-18,YSR and TRF profiles. Burlington: Department of Psychiatry,University of Vermont; 1991.

[34] Döpfner M, Schmeck K, Berner W, Lehmkuhl G, Poustka F. Zur Reliabilität und faktoriellen Validität der Child Behavior Checklist—eine Analyse in einer klinischen und einer Feldstichprobe [Reliability and factorial validity of the Child Behavior Checklist in a clinical and in a field sample]. Zeitschrift für Kinder- und Jugendpsychiatrie. 1994;22:189–205. 7975921

[35] Stiensmeier-Pelster J, Braune-Krickau M, Schürmann M, Duda K. DIKJ. Depressionsinventar für Kinder und Jugendliche [Depression Inventory for Children and Adolescents]. Göttingen: Hogrefe; 2014.

[36] Association AP. Diagnostic and statistical manual of mental disorders: DSM-5^™^ (5th ed.). Arlington, VA: American Psychiatric Publishing, Inc; 2013.

[37] Raven JC, Bulheller S, Häcker HO. Coloured Progressive Matrices (CPM). Göttingen: Hogrefe; 2001.

[38] Hair JF, Hult GTM, Ringle CM, Sarstedt M. A primer on partial least structural equation modeling (PLS-SEM). Los Angeles: Sage; 2014.

[39] Hair JF, Ringle CM, Sarstedt M. PLS-SEM: Indeed a silver bullet. Journal of Marketing Theory and Practice. 2011;19:139–51.

[40] Cohen J. Statistical power analysis for the behavioral sciences. Mahwah: Lawrence Erlbaum; 1988.

[41] Nunally JC, Bernstein I. Psychometric theory. New York: McGraw-Hill; 1994.

[42] Sarstedt M, Ringle CM. Treating unobserved heterogeneity in PLS path modeling: a comparison of FIMIX-PLS with different data analysis strategies. Journal of Applied Statistics. 2010;37(8):1299–318.

[43] Whitaker RC, Wright JA, Pepe MS, Seidel KD, Dietz WH. Predicting obesity in young adulthood from childhood and parental obesity. N Engl J Med. 1997;337(13):869–73. Epub 1997/09/26. doi: 10.1056/NEJM199709253371301 . 9302300 10.1056/NEJM199709253371301

[44] Galea S, Tracy M. Participation rates in epidemiologic studies. Ann Epidemiol. 2007;17(9):643–53. doi: 10.1016/j.annepidem.2007.03.013 . 17553702 10.1016/j.annepidem.2007.03.013

[45] Field AE, Austin SB, Taylor CB, Malspeis S, Rosner B, Rockett HR, et al Relation between dieting and weight change among preadolescents and adolescents. Pediatrics. 2003;112(4):900–6. . 14523184 10.1542/peds.112.4.900

[46] Allen G. Early Intervention: The next steps. London: Cabinet Office; 2011.

[47] van Strien T, Oosterveld P. The children's DEBQ for assessment of restrained, emotional, and external eating in 7- to 12-year-old children. Int J Eat Disord. 2008;41(1):72–81. doi: 10.1002/eat.20424 . 17634965 10.1002/eat.20424

[48] Hankey M, Kidwell KM, Nelson JM, Espy KA, Nelson TD. Weight Status as a Mediator of the Association Between Preschool Extraversion and Adolescent Restrained Eating. J Pediatr Psychol. 2017 doi: 10.1093/jpepsy/jsx049 . 28369620 10.1093/jpepsy/jsx049 PMC5896629

[49] Neimeijer RAM, Roefs A, Ostafin BD, de Jong PJ. Automatic Approach Tendencies toward High and Low Caloric Food in Restrained Eaters: Influence of Task-Relevance and Mood. Front Psychol. 2017;8:525 doi: 10.3389/fpsyg.2017.00525 ; 28443045 10.3389/fpsyg.2017.00525 PMC5387092

